# Quantifying diffuse airspace disease on portable chest radiographs in acute respiratory failure using the RALE score

**DOI:** 10.1186/s40635-026-00953-x

**Published:** 2026-07-28

**Authors:** Vanessa Gipson, Hafiz Qurashi, Pranav Jain, Neha Singh, Kyle Inman, Alicia N. Rizzo, Jacob Sinopoli, Taaha Mirza, Rimsha Ali, Nima Naghshtabrizi, Hanna Bobrysheva, Nanditha Venkatesan, Konstantin Golubykh, Niall Prendergast, Angela Hendricks, John-Paul Oliveria, Joshua Galanter, Julia Cluceru, Mohammadreza Negahdar, Caitlin Schaefer, Melissa Saul, Robin Joyce, Kevin Mitchell, Ellen Hughes, Bryan J. McVerry, Seyed Mehdi Nouraie, Georgios D. Kitsios

**Affiliations:** 1 Internal Medicine Residency Program, University of Pittsburgh, Pittsburgh, PA USA; 2 Division of General Internal Medicine, University of Pittsburgh, Pittsburgh, PA USA; 3 Division of Pulmonary, Critical Care, and Sleep Medicine, University of Pittsburgh Medical Center, UPMC Montefiore Hospital, 3459 Fifth Avenue, AllergyPittsburgh, PA NW62815213 USA; 4 Internal Medicine Residency Program, UPMC Central Pa, Harrisburg, PA USA; 5 Genentech, Inc., South San Francisco, CA USA; 6 Computer Vision Group, Veytel Inc., Pittsburgh, PA USA; 7 VA Pittsburgh Healthcare System, Pittsburgh, PA USA; 8 Department of Critical Care Medicine, University of Pittsburgh School of Medicine, Pittsburgh, PA USA; 9 Acute Lung Injury and Infection Center of Excellence, University of Pittsburgh, Pittsburgh, PA USA

**Keywords:** Airspace disease, Acute respiratory failure, RALE score, Chest X-ray, Acute respiratory distress syndrome

## Abstract

**Background:**

Portable chest radiographs (CXRs) obtained at presentation of acute respiratory failure (ARF) are interpreted qualitatively to assess airspace disease (ASD) and identify features consistent with acute respiratory distress syndrome (ARDS). The Radiographic Assessment of Lung Edema (RALE) score offers a semiquantitative measure of radiographic ASD, and has demonstrated prognostic value in ARDS, but its ability to quantify radiographic ASD burden across the full spectrum of ARF presentations remains uncertain.

**Methods:**

We analyzed 4,259 portable CXRs from 814 critically ill adults with expert‑adjudicated ARF subtypes, including ARDS, at‑risk for ARDS, cardiogenic pulmonary edema, acute exacerbation of interstitial lung disease (AE‑ILD), acute‑on‑chronic hypercapnic respiratory failure, and airway‑protection intubations. Trained clinicians, blinded to clinical data, assigned RALE scores. Analyses addressed (1) whether RALE distinguishes CXRs without ASD (airway‑protection intubations and acute-on-chronic hypercapnic respiratory failure) from those with any ASD, and (2) among CXRs with ASD, whether RALE quantifies diffuse bilateral opacification, distinguishing extensive from limited parenchymal involvement. Discrimination was evaluated using receiver operating characteristic curves, with sensitivity analyses assessing the influence of image‑quality features.

**Results:**

RALE scores increased stepwise from ASD absent to ASD limited to ASD diffuse, discriminated CXRs with any ASD from those without (area under the curve [AUC] 0.81, 95% confidence interval [CI] 0.77–0.85; rule‑out threshold RALE ≥ 7 yielded 96% sensitivity) and quantified diffuse bilateral opacification among ASD-positive CXRs with moderate accuracy (AUC 0.79, 95% CI 0.75–0.82). RALE distributions overlapped substantially across ARDS, cardiogenic pulmonary edema, and AE‑ILD, reflecting shared radiographic appearances rather than etiologic specificity. Suboptimal penetration inflated RALE scores and markedly reduced discrimination (AUC 0.59 vs 0.83 with adequate penetration).

**Conclusions:**

RALE quantifies radiographic ASD burden across the full spectrum of ARF at intubation — discriminating ASD-absent from ASD-present CXRs and stratifying the extent of parenchymal involvement among those with ASD. However, it did not distinguish between clinical syndromes that share similar diffuse radiographic presentations, highlighting the need to interpret RALE within the broader clinical context and ensure adequate image quality for accurate scoring. These findings reflect RALE’s nature as a radiographic burden metric rather than a diagnostic classifier.

**Supplementary Information:**

The online version contains supplementary material available at 10.1186/s40635-026-00953-x.

## Background

Portable chest radiographs (CXRs) are obtained routinely as part of standard care for patients with acute respiratory failure (ARF). These images are obtained across care settings, including the emergency department, hospital wards, and the intensive care unit (ICU), or following endotracheal intubation and are used to confirm appropriate positioning of placed devices and to assess lung parenchyma as part of the initial evaluation of ARF etiology. Clinicians interpret portable CXRs qualitatively to assess the presence, extent, and distribution of airspace disease (ASD), which informs diagnostic reasoning across ARF etiologies. This assessment contributes directly to syndromic classifications such as acute respiratory distress syndrome (ARDS) [1], cardiogenic pulmonary edema, and other causes of parenchymal ASD. Despite this central role in early clinical assessment, the burden of ASD on portable CXRs is not routinely quantified in a standardized manner, and interpretation remains largely qualitative and experience dependent.

The Radiographic Assessment of Lung Edema (RALE) score is a semiquantitative tool designed to measure the extent and density of alveolar opacities on frontal CXRs, originally developed in cohorts of patients with established ARDS. RALE divides the lungs into four quadrants and scores both the extent and density of opacification within each, yielding a total score (0–48) that captures the burden and distribution of ASD. This global assessment of radiographic severityhas shown associations with clinically meaningful outcomes in ARDS cohorts, including oxygenation, lung mechanics, and mortality [2, 3]. While manual RALE scoring is trainable and reproducible [4], it is impractical for widespread, real-time bedside use. Automated machine learning–based approaches that derive RALE scores [4, 5] or quantify radiographic opacities consistent with ARDS [6] have been proposed for prospective application when portable CXRs are obtained.

However, in early ARF, patients may present with radiographically normal lungs (ASD absent), limited opacities (ASD limited), or diffuse infiltrates (ASD diffuse) across a broad spectrum of clinical syndromes. This context raises two important clinical and research questions: *first, can RALE discriminate lungs without parenchymal abnormalities from those with any ASD?* Put differently, can RALE serve as a rule-out tool for essentially normal-appearing lungs, such as those of patients intubated for airway protection or with primary airway disease (e.g. status asthmaticus)? *Second, among CXRs exhibiting ASD, can RALE reliably quantify diffuse bilateral opacification, distinguishing extensive from limited parenchymal involvement?*

Addressing these questions would clarify RALE’s utility in early evaluation of ARF beyond cohorts with confirmed ARDS [3, 7, 8].

Moreover, portable CXRs obtained in critically ill patients vary substantially in technical quality, including differences in penetration, positioning, and the presence of artifacts – factors that may influence apparent opacification and, by extension, RALE scoring [9]. However, the influence of image acquisition features on RALE scoring remains poorly defined.

To address these knowledge gaps, we analyzed RALE scores on portable CXRs obtained immediately post-intubation in a large, prospective cohort of patients with ARF. Our objectives were to evaluate whether RALE can (1) discriminate ASD-absent from ASD-present CXRs, (2) quantify the extent of bilateral opacification among ASD-positive CXRs, and (3) characterize the influence of image acquisition quality on RALE distributions and discriminative performance.

## Methods

### Study population

We analyzed 814 critically ill, intubated adult patients with ARF enrolled in the Acute Lung Injury Registry (ALIR) and Biospecimen Repository at UPMC Hospitals between 2011 and 2024. These patients contributed 4,259 frontal CXRs. Detailed cohort characteristics have been described previously [10–12].

### ARF clinical classifications

A consensus committee of attending pulmonary and critical care physicians with subspecialty board certification and active clinical ICU practice reviewed all available clinical, radiographic, laboratory, and physiologic data at the time of enrollment. The adjudication methodology has been previously described [12]. All committee members routinely interpret portable CXRs and apply ARDS diagnostic criteria as part of standard clinical care. Each participant was classified into one of the following non-overlapping categories (clinical subtypes) [13]:

1. ARDS per Berlin Criteria [1]
2. At- Risk for ARDS: identifiable risk factor(s) for acute lung injury (Table S1) but not fulfilling ARDS diagnostic criteria.
3. Congestive Heart Failure (CHF): hypoxemic ARF from cardiogenic pulmonary edema.
4. Airway Controls: intubation for airway protection without primary pulmonary pathology.
5. Acute on Chronic Hypercapnic Respiratory Failure (AoCHRF): acute decompensation of chronic ventilatory failure from obstructive airways disease.
6. Acute Exacerbation of Interstitial Lung Disease (AE-ILD): acute worsening of ILD with diffuse radiographic abnormalities.
7. Other/Multifactorial: Patients not clearly fitting other categories or with overlapping features from multiple subtypes.

Inter-rater agreement for these classifications was highest for subtypes representing radiographic extremes: airway controls (92.9% unanimous agreement), ARDS (84.5%), and AE-ILD (87.5%) (Figure S1) [12]. Lower agreement was observed for subtypes with intermediate or overlapping features, particularly CHF (50%) and Other/Multifactorial (16.7%). This adjudication process incorporated a qualitative review of portable CXRs and/or CT imaging obtained at enrollment, including assessment of bilateral ASD when applicable, consistent with ARDS (“ARDS-like” radiographic presentation). No quantitative radiographic scoring was performed during adjudication; committee members assigned clinical subtypes using standard diagnostic criteria and qualitative radiographic assessment only, distinct from the independent quantitative RALE scoring performed subsequently by trained raters blinded to all clinical and subtype data. CT imaging was reviewed as part of adjudication when available and clinically obtained (30.1%); the adjudication protocol did not mandate CT for all patients. This blinded adjudication and blinded RALE scoring minimized ascertainment bias in evaluating RALE’s discriminative performance.

The committee members also adjudicated for the presence of ARDS risk factors as per the Berlin definition criteria [14], independent of whether ASD was present. Risk factor types were categorized as:

1. Direct risk factor: Primary pulmonary insults (e.g., pneumonia, aspiration, lung contusion, inhalational injury).
2. Indirect risk factor: extra-pulmonary insults (e.g., non-pulmonary sepsis, pancreatitis, massive transfusion, severe trauma).
3. Mixed risk factors: both direct and indirect risk factors present.

### Airspace disease (ASD) classification and operational definitions

Our primary analytic goal was to evaluate RALE’s ability to answer two clinically relevant questions in early ARF:

1. Presence of parenchymal disease: Does a given CXR show any evidence of airspace disease, or are the lungs essentially normal (ASD absent)?
2. Extent of opacification among CXRs with ASD: Among CXRs with ASD, does the image exhibit diffuse bilateral opacification (ASD diffuse), or is parenchymal involvement more limited (ASD limited)?

**Fig. 1:**
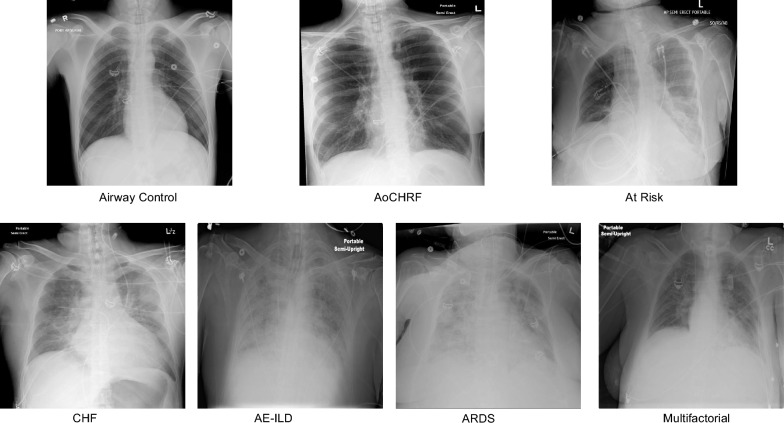
Representative index CXRs for each adjudicated ARF clinical subtype: Airway Control, AoCHRF, At Risk, CHF, AE-ILD, ARDS, and Other/Multifactorial. Images illustrate the expected radiographic spectrum, from minimal or absent parenchymal abnormality (Airway Control) to bilateral diffuse airspace opacities (ARDS, AE-ILD, CHF). All images are frontal portable CXRs

To operationalize these questions, we used the expert-adjudicated ARF clinical subtypes as the reference standard for expected radiographic appearance. These ASD categories reflect the radiographic opacification patterns that are definitionally expected for each clinical syndrome; they do not represent an independent imaging reference standard. For example, a patient intubated for airway protection without evidence of pneumonia or consolidation would be expected to have essentially normal lungs (ASD absent) and low/normal RALE scores, whereas a patient with cardiogenic pulmonary edema, as adjudicated by the expert panel, would be expected to have bilateral diffuse airspace opacities (Fig. 1shows representative index CXRs across adjudicated clinical subtypes).

Based on these reference standards, we derived three ASD categories:

- ASD Absent: Airway Controls and AoCHRF—syndromes expected to have minimal or no parenchymal abnormalities.
- ASD Limited: At-Risk for ARDS—patients with ARDS risk factors but lacking the bilateral diffuse opacities characteristic of ARDS.
- ASD Diffuse: ARDS, CHF, AE-ILD, and Other/Multifactorial—syndromes defined by the presence of bilateral diffuse opacities.

For analysis, we applied a binary classification aligned with the first question: ASD absent versus ASD present (limited + diffuse). The second question was addressed by comparing ASD diffuse versus ASD limited among CXRs with ASD present.

### CXR repository creation and index CXR selection

All frontal CXRs obtained during the ICU admission for enrolled patients were curated into a de-identified repository (Pulmo-Annotator, Veytel, Inc) [4, 15]. For each patient, the index CXR was defined as the first frontal CXR obtained on the calendar day post endotracheal intubation. The index CXR was selected to mirror real-world application of RALE-based algorithms, i.e., by scoring the first CXR post-intubation when ARF etiology and clinical subtype classification may be uncertain. All remaining ICU CXRs were retained for secondary image-level analyses of RALE distributions and associations with acquisition features.

RALE scoring followed the original description [2] and a standardized multi-rater protocol developed and validated in our group [4]. Each CXR was divided into four quadrants; raters assigned quadrant-level extent (0–4, reflecting percentage of quadrant with opacification) and density (0–3, reflecting opacity severity) scores. Quadrant scores (extent*density) were summed across all four quadrants to yield a total RALE score (0–48). Fifteen clinicians (interns, residents, fellows, and attendings) completed structured training anchored to an expert reviewer (GDK). After training, raters independently scored all study CXRs while blinded to clinical data, adjudicated subtype, and outcomes. Three raters (G.D.K., N.P., A.R.) also participated in the adjudication committee; both processes were conducted with mutual blinding, with RALE scoring performed after adjudication and raters blinded to subtype assignments throughout. We calculated the mean RALE score from trained raters’ scores. Inter-rater reliability for this multi-rater protocol has been recently described [4], with an overall intraclass correlation coefficient of 0.92 (95% CI 0.89–0.94) across trained raters, indicating excellent agreement.

### Qualitative features of CXR images

An expert reviewer (GDK) assessed standardized image quality features for all CXRs to examine their influence on RALE scoring:

1. Penetration (adequacy): assessed by identifying the deepest visible spinal landmark, categorized from caudal to cranial as: all vertebral bodies, diaphragm, heart, carina, or only the thoracic inlet visible. For analysis, penetration was dichotomized as adequate (all vertebral bodies, diaphragm, or heart visible) versus suboptimal (only carina or thoracic inlet visible).
2. Artifacts: presence of any major artifacts obscuring lung parenchyma (e.g., defibrillator pads, monitor leads, external tubing). The reviewer annotated artifact presence on each image, and for analysis, we summarized artifacts as the total number of artifact annotations per image (artifact count). For stratified discrimination analyses, artifacts were additionally dichotomized as any vs none.
3. Overall Image Quality: global assessment categorized as good vs poor, with “poor” indicating limited interpretability and potential need for repeat imaging (e.g., incomplete field of view, patient rotation, positioning limitations) (Figure S2).

### Statistical analysis

We first characterized RALE Score distributions on the index CXR across ARF subtypes, ASD categories (absent, limited, diffuse), and presence/absence of ARDS risk factors. RALE distributions were summarized using medians and interquartile ranges (IQR) and compared across groups using nonparametric tests with adjustment for multiple comparisons. To evaluate the ability of RALE to discriminate ASD presence, we fit a logistic regression model with ASD status (present vs absent) as the dependent variable and index RALE (continuous, 0–48) as the sole predictor. Discrimination was summarized using the area under the receiver-operating curve (AUC) with DeLong 95% confidence intervals. From this model, we derived two integer RALE thresholds: (1) a rule-out threshold optimized for high sensitivity and (2) a balanced threshold maximizing Youden’s index (sensitivity + specificity –1). For each threshold, we calculated sensitivity, specificity, positive and negative predictive values, likelihood ratios, and confusion matrices. We performed internal validation using tenfold stratified cross-validation, with stratification preserving the proportion of ASD presence/absence in each fold. As a pre-specified sensitivity analysis, we re-evaluated RALE discrimination of ASD diffuse versus ASD limited after excluding patients in the Other/Multifactorial category from the ASD diffuse group, to assess the robustness of the primary findings to inclusion of the most diagnostically ambiguous subgroup. In secondary analyses, we examined the association between RALE and image quality features using a multivariable linear mixed-effects model with random intercepts by SubjectID, with RALE (0–48) as the outcome and penetration (suboptimal vs adequate), artifact burden (count), and overall image quality (poor vs good) as predictors, to account for within-patient correlation across repeated CXRs. We then assessed the stability of discrimination and threshold performance after stratification by image quality features. Exploratory analyses incorporating age and body mass index (BMI) are reported in the Supplement. RALE scores and ASD classification were complete for all 814 index CXRs; no imputation was performed for primary analyses. For image-level secondary analyses, acquisition feature annotations were available for 4,258 of 4,259 CXRs (one image lacked penetration annotation and was excluded from image-level regression analyses); no imputation was performed.

### Statistical software

All analyses were conducted in R (version 4.4.3). Packages included tidyverse/dplyr for data management, pROC for ROC/AUC and DeLong CIs, lme4 and lmerTest for linear mixed-effects modeling, car for partial R^2^ and variance diagnostics, and ggplot2 for visualization. Reporting adheres to STROBE guidelines for observational studies.

## Results

### Cohort characteristics

**Fig. 2:**
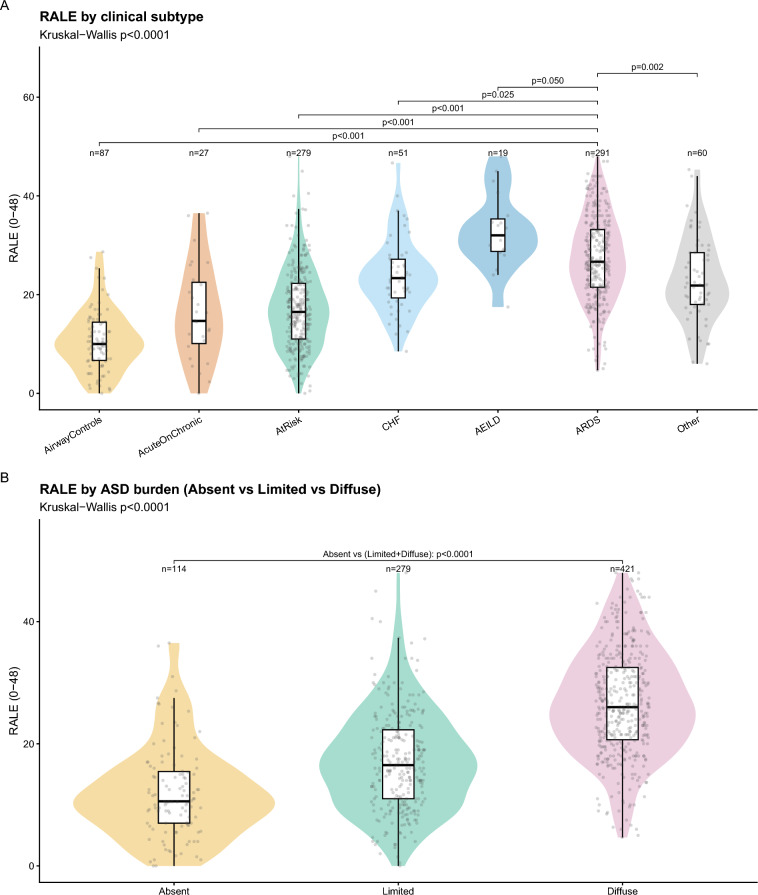
RALE on the index CXR among intubated patients, by clinical subtype and airspace disease (ASD) burden classification (**A**) Distribution of RALE scores by adjudicated ARF clinical subtype on index CXR. (**B**) RALE scores by ASD burden stratum (absent, limited, diffuse). Violin plots show score distributions; boxplots show median (IQR); points represent individual patients. Group sizes are shown above each category. Overall differences were assessed with Kruskal–Wallis testing; displayed ARDS-anchored p-values in Panel A are from Dunn tests with Benjamini–Hochberg adjustment, and the displayed p-value in Panel B reflects the comparison of ASD Absent versus ASD present (limited + diffuse). RALE range 0–48

We analyzed 814 critically ill, intubated adults with expert-adjudicated classification of ARF subtypes, contributing 4,259 CXRs (Table 1). The cohort had a median age of 59 years (IQR 47–68), 57% were male, and a median BMI of 30 (IQR 26–36). On the index CXR, clinical subtypes included airway controls (n = 87), AoCHRF (n = 27), at-risk for ARDS (n = 279), CHF (n = 51), AE-ILD (n = 19), ARDS (n = 291), and other/multifactorial (n = 60) (Fig. 2A). ASD was present in 700 of 814 patients (86%), reflecting the lung-injury-enriched enrollment design of the ALIR cohort. Using post-hoc ASD stratification based on these clinical subtypes, 114 patients were classified as ASD absent (airway controls and AoCHRF), 279 as ASD limited (at-risk for ARDS), and 421 as ASD diffuse (ARDS, CHF, AE-ILD, and other). Baseline demographics and clinical characteristics stratified by ASD category are presented in Table 1. The worst PaO₂/FiO₂ ratio in the first 24 h decreased stepwise across ASD categories (ASD absent: 202 [143–240] vs. ASD limited: 170 [117–237] vs. ASD diffuse: 127 [80–187] mmHg; p < 0.001), and 60-day mortality increased from 21% in ASD absent to 39% in ASD diffuse (p < 0.001), providing face validity for the ASD burden stratification.

**Table 1:** Baseline characteristics of the index CXR cohort, stratified by airspace disease (ASD) burden

**Variable**	**Absent** (n = 114)	**Limited** (n = 279)	**Diffuse** (n = 421)	**Overall** (n = 814)	P-value*
Age, median [IQR], years	56 [44, 66]	61 [51, 70]	58 [46, 67]	59 [47, 68]	0.003
Male, N (%)	61 (54%)	166 (59%)	241 (57%)	468 (57%)	0.500
BMI median [IQR]	28 [25, 32]	29 [25, 36]	31 [26, 37]	30 [26, 36]	< 0.001
**Race, (N (%))**					0.607
White	101 (89%)	248 (89%)	375 (89%)	724 (89%)	
Black	12 (11%)	28 (10%)	36 (9%)	76 (9%)	
**History of chronic disease**					
COPD, N (%)	27 (24%)	72 (26%)	67 (16%)	166 (20%)	0.004
Chronic kidney disease, N (%)	13 (11%)	49 (18%)	70 (17%)	132 (16%)	0.300
Diabetes mellitus, N (%)	27 (24%)	116 (42%)	136 (32%)	279 (34%)	0.001
Immunosuppression, N (%)	18 (16%)	40 (14%)	114 (27%)	172 (21%)	< 0.001
**Mechanical ventilation parameters**					
Worst PaO2/FiO2 in first 24 h, median [IQR], mmHg	202 [143, 240]	170 [117, 237]	127 [80, 187]	157 [94, 205]	< 0.001
Driving pressure, median [IQR] (cm H₂O)	11 [8, 15]	12 [9, 16]	14 [11, 18]	13 [10, 17]	< 0.001
**Outcomes**					
VFD, median [IQR], days	25 [19, 27]	21 [7, 25]	15 [0, 24]	20 [0, 25]	< 0.001
60-day mortality, N (%)	24 (21%)	84 (30%)	165 (39%)	273 (34%)	< 0.001

*P values were calculated using the Kruskal–Wallis or Chi-2 test

Values are reported as median [interquartile range, IQR] for continuous variables and n (%) for categorical variables. P-values compare ASD Absent vs Limited vs Diffuse using Kruskal–Wallis tests for continuous variables and Pearson’s chi-squared tests for categorical variables

ASD, airspace disease; BMI, body mass index; COPD, chronic obstructive pulmonary disease; CXR, chest radiograph; PaO₂/FiO₂, ratio of arterial oxygen tension to fraction of inspired oxygen; VFD, ventilator-free days.

ASD categories were derived from expert-adjudicated clinical subtypes:

**ASD Absent:** airway controls and acute-on-chronic hypercapnic respiratory failure (AoCHRF), syndromes with minimal or no parenchymal abnormalities.

**ASD Limited:** patients at risk for ARDS, with identifiable risk factors but lacking bilateral diffuse opacities.

**ASD Diffuse:** patients with ARDS, cardiogenic pulmonary edema (CHF), acute exacerbation of interstitial lung disease (AE-ILD), or other/multifactorial causes, characterized by bilateral diffuse opacities.

RALE distributions across ARF clinical subtypes:

On the index CXR, RALE differed significantly across ARF clinical subtypes (Kruskal–Wallis p < 0.0001; Fig. 2A). Median RALE scores (IQR) increased in the expected clinical severity gradient: lowest in airway controls 10.0 (6.7–14.4) and AoCHRF 14.7 (10.1–22.5); intermediate in at-risk for ARDS 16.5 (11.0–22.3); and higher in other/multifactorial 21.8 (18.0–28.5) and CHF 23.3 (19.3–27.2), with ARDS 26.7 (21.5–33.2) and AE-ILD 32.0 (28.8–35.3) demonstrating the highest values (Fig. 2A). ARDS-anchored pairwise comparisons are displayed in Fig. 2A.

When stratified by ASD burden, median RALE (IQR) increased stepwise from ASD absent 10.6 (7.0–15.5) to ASD limited 16.5 (11.0–22.3) to ASD diffuse 26.0 (20.7–32.5) (Kruskal–Wallis p < 0.0001; Fig. 2B). In the binary classification, median RALE (IQR) was higher in ASD present (n = 700) 22.4 (16.0–29.3) than ASD absent (n = 114) 10.6 (7.0–15.5) (Wilcoxon rank-sum p < 0.0001; Figure S3).

### RALE by ARDS risk factor category

**Fig. 3:**
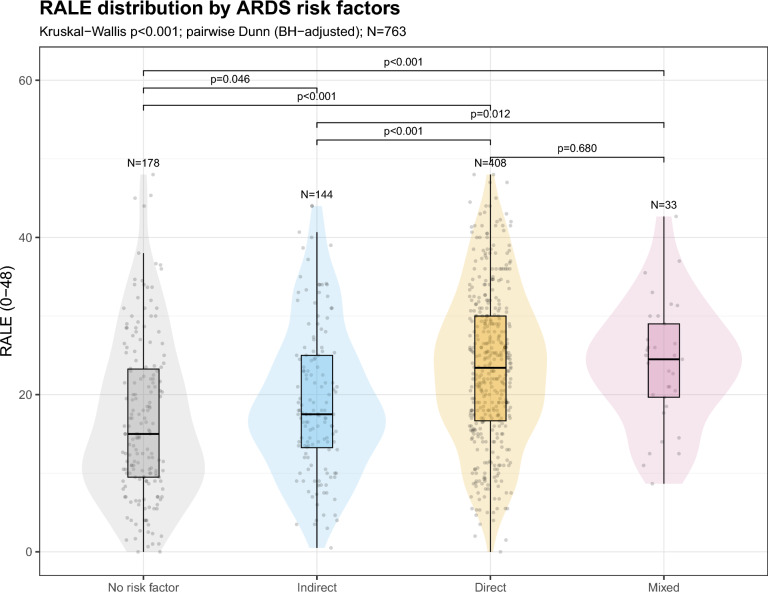
RALE distribution by ARDS risk factor category on the index CXR: Index CXR RALE scores by ARDS risk factor category (direct, indirect, mixed, or none) among patients excluding the CHF clinical subtype (n = 763). Points show individual patients; boxplots show median (IQR). Overall differences were assessed with Kruskal–Wallis testing; pairwise comparisons use Dunn testing with Benjamini–Hochberg adjustment (as displayed)

On the index CXR, RALE differed across ARDS risk factor categories (Kruskal–Wallis p < 0.0001; Fig. 3). In BH-adjusted pairwise comparisons, RALE was higher in the indirect (p = 0.046), direct (p < 0.001), and mixed (p < 0.001) risk factor groups than in the no risk factor group. Among risk factor categories, RALE was higher in the direct group than in the indirect group (p < 0.001) and in the mixed group than in the indirect group (p = 0.012), whereas the direct and mixed groups did not differ (p = 0.680).

### Discrimination of ASD by the index RALE score

**Fig. 4:**
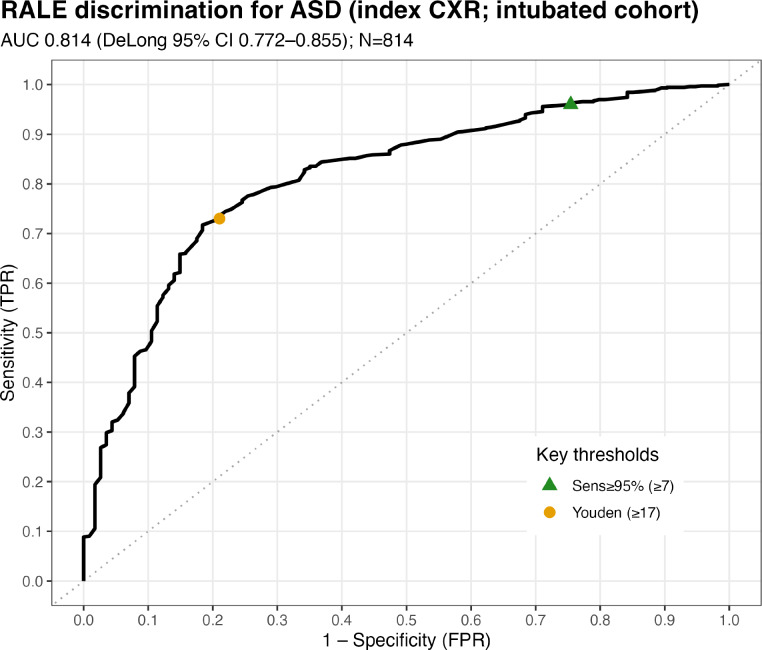
Discrimination of ASD presence using index CXR: RALE Receiver operating characteristic (ROC) curve showing the ability of the index CXR RALE score (0–48) to distinguish lungs with any airspace disease (ASD present) from those without ASD (ASD absent; airway controls + AoCHRF). The area under the curve (AUC) was 0.814 (DeLong 95% CI, 0.772–0.855; N = 814). Markers indicate two cohort-derived thresholds: a rule-out threshold (RALE ≥ 7) optimized for high sensitivity (≥ 95%) to identify any parenchymal abnormality, and a balanced threshold (RALE ≥ 17) maximizing Youden’s index, offering an equilibrium between sensitivity and specificity

**Fig. 5:**
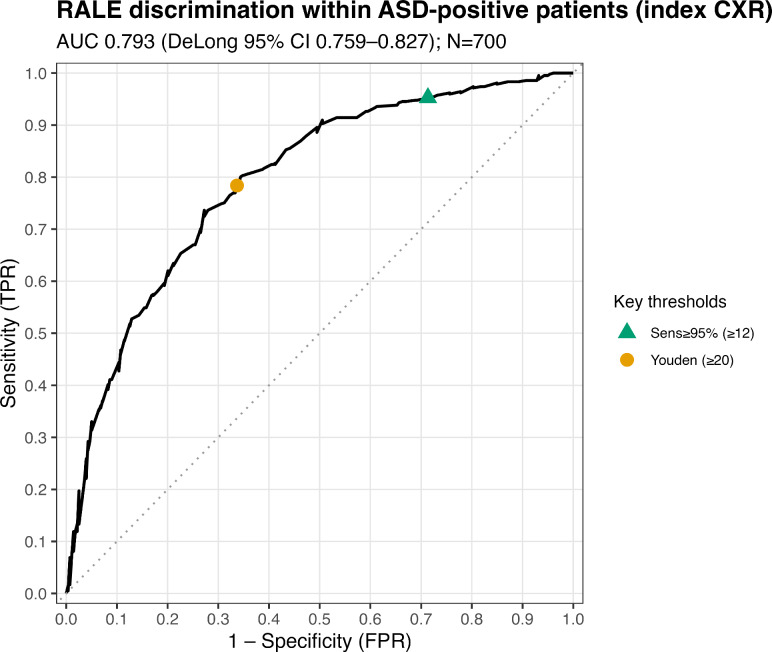
Discrimination of diffuse versus limited airspace disease (ASD) among ASD-positive patients using RALE on index CXR: Receiver operating characteristic (ROC) curve showing the ability of the index CXR RALE score (0–48) to distinguish ASD diffuse (lungs with bilateral, extensive opacities consistent with diffuse parenchymal disease) from ASD limited (lungs with mild or focal opacities) among patients with any ASD (N = 700). The area under the curve (AUC) was 0.793 (DeLong 95% CI, 0.759–0.827). Markers indicate two cohort-derived thresholds: a rule-out threshold (RALE ≥ 12) optimized for high sensitivity (≥ 95%) to identify diffuse opacities, and a balanced threshold (RALE ≥ 20) maximizing Youden’s index to balance sensitivity and specificity

RALE discriminated ASD present from ASD absent (binary classification aligned with the first key question: *can RALE distinguish ASD-absent from ASD-present CXRs?*) with an AUC of 0.814 (95%CI, 0.772–0.855; N = 814) (Fig. 4). Using cohort-derived thresholds, a rule-out threshold of 7 achieved sensitivity 0.96 (0.94–0.97) and specificity 0.29 (0.21–0.38), whereas a balanced threshold of 17 yielded sensitivity 0.72 (0.68–0.75) and specificity 0.82 (0.73–0.88) (Table 2). Ten-fold stratified cross-validation demonstrated good calibration (Figure S4), with confusion matrices provided in Table S2. Among patients with ASD present (n = 700), RALE discriminated ASD diffuse vs limited (aligned with the second key question: *among ASD-present CXRs, can RALE quantify the extent of bilateral opacification?*) with an AUC of 0.793 (95% CI 0.759–0.827; Fig. 5). A rule-out threshold of 12 for ASD diffuse achieved sensitivity 0.95 and specificity 0.29; a balanced threshold of 20 yielded sensitivity 0.78 and specificity 0.66 (Table S3). In a pre-specified sensitivity analysis excluding Other/Multifactorial patients (n = 60) from the ASD diffuse group, RALE discrimination of ASD diffuse versus ASD limited yielded an AUC of 0.809 (95% CI 0.775–0.843; N = 640), consistent with the primary analysis (AUC 0.793), indicating that findings were not driven by the inclusion of the most diagnostically ambiguous subgroup in the ASD diffuse category.

**Table 2:** Operating characteristics of cohort-derived RALE thresholds on the index CXR for discriminating ASD present versus ASD absent

Threshold	RALE cutoff	Sensitivity (95% CI)	Specificity (95% CI)	PPV (95% CI)	NPV (95% CI)	LR + (95% CI)	LR − (95% CI)
Youden	17	0.72 (0.68–0.75)	0.82 (0.73–0.88)	0.96 (0.94–0.97)	0.32 (0.27–0.38)	3.89 (2.64–5.74)	0.35 (0.30–0.40)
Rule-out (Sens ≥ 95%)	7	0.96 (0.94–0.97)	0.29 (0.21–0.38)	0.89 (0.87–0.91)	0.52 (0.39–0.64)	1.35 (1.20–1.51)	0.15 (0.10- 0.24)

Sensitivity, specificity, positive predictive value (PPV), negative predictive value (NPV), positive likelihood ratio (LR +), and negative likelihood ratio (LR −) are shown for two integer RALE thresholds applied as RALE ≥ cutoff on the index CXR. The Youden cutoff maximizes Youden’s J; the rule-out cutoff is the lowest integer RALE achieving sensitivity ≥ 95%. Values are point estimates with 95% confidence intervals

### Image acquisition features and RALE magnitude

**Fig. 6:**
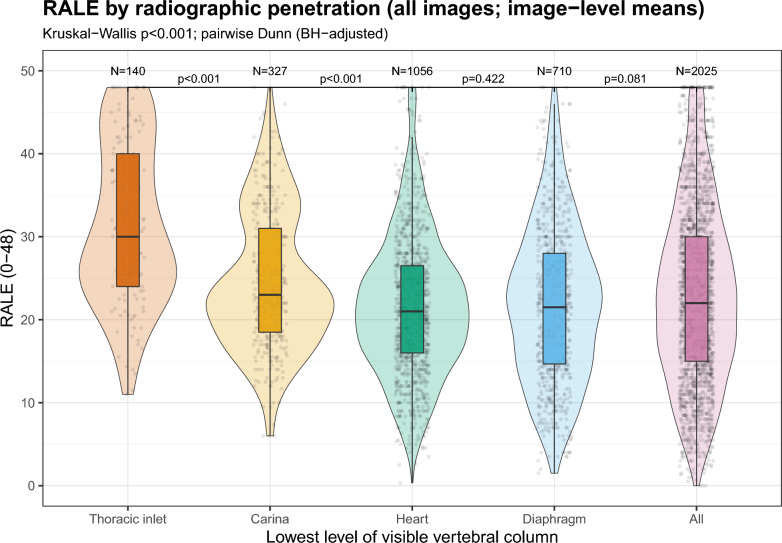
RALE by radiographic penetration across all CXRs: Violin plots show the distribution of RALE (0–48) across penetration categories defined by the lowest visible vertebral level (thoracic inlet, carina, heart, diaphragm, or all vertebral bodies). Overall differences were assessed with Kruskal–Wallis testing, with adjacent pairwise comparisons performed using Dunn tests with Benjamini–Hochberg adjustment; N for each category is shown above the violins

RALE differed across radiographic penetration categories defined by the lowest visible vertebral level (Kruskal–Wallis p < 0.0001; Fig. 6). BH-adjusted Dunn testing showed a stepwise decrease in RALE from thoracic inlet to carina (p < 0.0001) and from carina to heart (p < 0.0001), followed by a plateau across the lower penetration categories (heart vs diaphragm p = 0.422; diaphragm vs all p = 0.081) (Fig. 6).

**Fig. 7:**
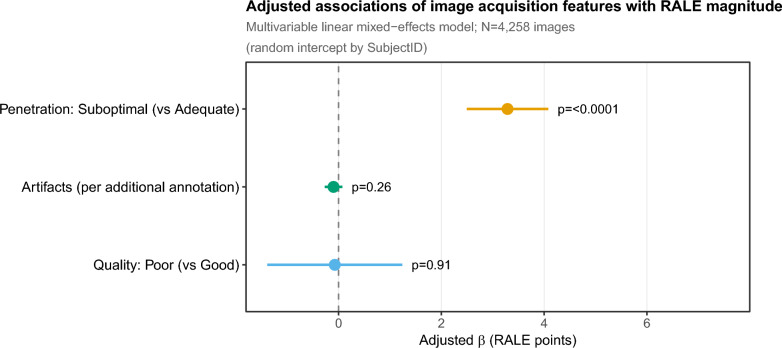
Adjusted associations of image acquisition features with RALE magnitude: Forest plot from a multivariable linear mixed-effects model evaluating associations between image acquisition characteristics and RALE score (0–48) across all CXRs (N = 4,258), with random intercepts by SubjectID. Points represent adjusted regression coefficients (β, RALE points) and horizontal bars represent 95% confidence intervals; the dashed vertical line denotes no association (β = 0). Artifact burden is modeled per additional annotation

In a multivariable image-level linear mixed-effects model with random intercepts by SubjectID, suboptimal penetration (vs adequate) was associated with higher RALE scores (+ 3.29 points; 95% CI 2.50–4.08; p < 0.0001); poor overall image quality (vs good) and artifact count were not independently associated with RALE scores (image quality: β − 0.07, 95% CI − 1.39 to 1.24, p = 0.91; artifact count: β − 0.10, 95% CI − 0.27 to 0.07, p = 0.26) (Fig. 7; Table S4).

### Discrimination performance by image acquisition features

RALE discrimination varied substantially by image acquisition features (Table 3). With adequate penetration, discrimination was good (AUC 0.83, 95% CI 0.79–0.87), but it was substantially reduced with suboptimal penetration (AUC 0.59, 95% CI 0.37–0.80). Discrimination estimates were similar for good-quality (AUC 0.81, 95% CI 0.77–0.86) and poor-quality CXRs (AUC 0.79, 95% CI 0.46–1.00), though the poor-quality sample was small. Discrimination was higher when artifacts were absent (AUC 0.85, 95% CI 0.79–0.91) than when any artifact was present (AUC 0.78, 95% CI 0.72–0.84). Exploratory inclusion of age and BMI did not materially improve discrimination (Table S5).

**Table 3:** Diagnostic performance of RALE for ASD status, stratified by image acquisition features on the index CXR

Stratum	N	AUC (95% CI)	Rule-out cutoff (RALE)	Youden cutoff (RALE)
**Penetration**				
Adequate	730	0.83 (0.79–0.87)	7	17
Suboptimal	84	0.59 (0.37–0.80)	11	26
**Image Quality**				
Good	794	0.81 (0.77–0.86)	7	17
Poor	20	0.79 (0.46–1.00)	8	11
**Artifacts**				
None	410	0.85 (0.79–0.91)	8	16
Any	404	0.78 (0.72–0.84)	7	15

Area under the ROC curve (AUC; DeLong 95% CI) and cohort-derived integer RALE cut points are shown within each imaging stratum; N indicates the number of index CXR with available acquisition features present. Penetration was categorized as adequate (lowest visible vertebral level: all/diaphragm/heart) versus suboptimal (carina/thoracic inlet). Overall image quality was categorized as good versus poor. Artifacts were categorized as none versus any. The rule-out cutoff was defined as the lowest integer RALE achieving sensitivity ≥ 0.95 within the stratum; the Youden cutoff maximized Youden’s J within the stratum

## Discussion

We evaluated RALE scoring in a prospective cohort representing the full spectrum of ARF at intubation, extending beyond prior work in ARDS populations. RALE quantified radiographic ASD burden and discriminated ASD presence with moderate accuracy (AUC 0.81), with findings reflecting RALE’s ability to align with the radiographic opacification patterns expected from clinical syndrome classifications rather than independently resolve etiologic diagnosis. Image acquisition quality, particularly penetration, substantially influenced both RALE magnitude and discriminative performance, underscoring the need for quality-aware implementation.

Most prior RALE studies have focused on established ARDS or COVID-19 pneumonia, where bilateral diffuse opacities are present by definition [2, 3, 7, 8, 15, 16]. We extended this work by evaluating RALE at the time of intubation, when ARF etiology may be uncertain. Our cohort included not only ARDS but also at-risk states, CHF, airway controls and other clinical presentations, yielding graded RALE distributions that tracked with radiographic burden rather than specific etiologies. While prior ARDS-enriched cohorts demonstrated excellent discrimination for ARDS diagnosis (AUC 0.91) [7], our inclusive cohort showed more modest discrimination between ASD diffuse and ASD limited (AUC 0.79) because multiple syndromes, such as ARDS, cardiogenic edema, and AE-ILD, share similar diffuse opacities. This overlap is not misclassification; rather, it reflects RALE’s design as a measure of radiographic pattern rather than etiology. RALE reliably identifies lungs with diffuse opacities but cannot distinguish the underlying cause, underscoring the need for clinical context alongside radiographic assessment.

Two pragmatic thresholds emerged: a rule-out threshold (RALE ≥ 7; sensitivity 0.96) optimized to exclude significant ASD, and a balanced threshold (RALE ≥ 17; sensitivity 0.72, specificity 0.82, LR + 3.89) that balances false-positive and false-negative rates, suited for settings requiring higher specificity, such as clinical trial enrichment or automated alerts. It is important to recognize that both thresholds function as adjuncts to clinical evaluation rather than standalone diagnostic tests. At the observed ASD prevalence of 86% in our cohort, the NPV of the rule-out threshold (0.52) is insufficient for clinical rule-out, reflecting the high pre-test probability of ASD in this cohort; Figure S5 illustrates expected PPV and NPV across a range of ASD prevalence, demonstrating improvement at lower pre-test probability settings. These threshold values are broadly consistent with prior literature; the rule-out threshold of RALE ≥ 7 aligns directionally with prior work identifying a RALE score of 10 as diagnostically informative for ARDS [7]. Both thresholds remained stable under cross-validation. Adding age and BMI to RALE-based models did not meaningfully improve discrimination, suggesting that RALE captures radiographic burden relatively independently of patient characteristics, though whether combining RALE with physiologic variables (e.g., oxygenation, respiratory mechanics) would enhance classification warrants further investigation.

Suboptimal penetration—common in portable ICU radiographs—was associated with higher RALE values and markedly attenuated discrimination (AUC 0.83 with adequate penetration vs. 0.59 with suboptimal penetration). The mechanism is well-established: underpenetrated CXRs increase apparent parenchymal density, creating artifactual opacification [9]. In contrast, poor overall image quality and artifact burden were not independently associated with RALE scores in mixed-effects models accounting for withing-patient correlation. These findings provide the empirical basis for a quality-adjusted RALE framework: scores should be interpreted — and operating thresholds applied — in the context of penetration adequacy, with penetration-specific thresholds (Table 3) used when image quality data are available.

Multiple groups have developed machine learning approaches to automate RALE scoring or derive similar radiographic severity measures [5, 6], enabling scalable deployment when portable CXRs are obtained. Our results provide important context for such systems: even human expert scoring with standardized protocols is affected by acquisition quality. Automated systems must explicitly address this challenge through quality-aware training, preprocessing to normalize penetration, quality flagging, or restriction of analysis to adequately penetrated images. Both automated and human-scored RALE require interpretation in the context of image quality to avoid systematic bias.

Study strengths include prospective enrollment, blinded expert adjudication, standardized multi-rater RALE scoring with mutual blinding between scoring and adjudication processes, and systematic evaluation of image acquisition features. However, several limitations warrant consideration. First, this single-center cohort was enriched for lung injury syndromes by design and evolving enrollment priorities [12], resulting in high ASD prevalence (86%) that does not reflect true ICU population prevalence. This spectrum bias limits the transportability of derived thresholds: PPV and NPV are prevalence-dependent and will differ substantially in settings with lower ASD burden, such as community hospitals, emergency departments, or non-intubated patient populations. External validation in lower-prevalence populations is necessary before these thresholds can be broadly deployed. Second, a fundamental methodological consideration is that the ASD classification framework was derived from expert-adjudicated clinical subtypes whose definitions incorporate qualitative radiographic assessment — most notably, the bilateral infiltrate criterion required for ARDS per Berlin criteria. The study therefore evaluates RALE’s ability to quantify radiographic patterns definitionally expected to differ across these categories, rather than performance against a fully independent imaging reference standard. Clinical adjudication was performed without access to quantitative RALE scores, mitigating the most operationally serious form of circularity, but both the reference standard and the index test ultimately reflect lung radiographic appearance. As the clinical reference standard assigns categorical syndrome labels rather than continuous radiographic burden measurements, the study provides construct validity evidence that RALE scores correspond to expected opacification patterns across clinical categories, rather than a direct validation of RALE as a burden quantification tool against an independent radiographic comparator; CT-based quantification of parenchymal opacification would provide the most appropriate such reference standard for future validation studies. Third, three of the fifteen trained RALE raters also participated in the adjudication committee; although both processes were conducted with mutual blinding, residual contextual knowledge transfer from adjudication to RALE scoring in these individuals cannot be fully excluded. Fourth, we used consensus RALE (mean across trained raters) as our primary measure. Individual raters, untrained readers, or fully automated systems may show different performance characteristics. Fifth, penetration and other image quality features were assessed subjectively by the expert reviewer. While this approach ensured consistent classification, more objective penetration measures (e.g., gray-scale histogram features, automated spinous process detection) might reduce measurement error and facilitate automated quality assessment. Finally, clinical subtype classification represents ARF syndrome at the time of index evaluation. We did not examine longitudinal transitions between subtypes or subsequent ARDS development. The study evaluated discrimination of ASD presence at a single time point rather than longitudinal RALE trajectories or subtype evolution.

## Conclusion

In conclusion, RALE quantifies radiographic ASD burden across the full spectrum of ARF at intubation — discriminating ASD-absent from ASD-present CXRs and stratifying the extent of parenchymal involvement — but cannot distinguish between clinical etiologies that share similar radiographic morphology. These findings establish RALE as a radiographic burden metric rather than a diagnostic classifier, and provide pragmatic operating points for quality-aware implementation in automated post-intubation screening and clinical trial enrichment. External validation in lower-prevalence and non-ICU settings is necessary before these thresholds can be broadly deployed.

*Take Home Message:* In this prospective cohort study of 814 critically ill patients, RALE quantified radiographic ASD burden across the full spectrum of ARF at intubation, discriminating CXRs without airspace disease from those with ASD and stratifying the extent of parenchymal involvement. RALE distributions overlapped substantially across syndromes sharing diffuse radiographic morphology, establishing RALE as a measure of radiographic burden rather than an etiologic discriminator. Pragmatic, quality-stratified operating thresholds are provided to guide implementation in automated screening and clinical trial enrichment.

## Supplementary Information

Additional file 1.

## Data Availability

The de-Identified individual participant data supporting the findings of this study are available from the corresponding author upon reasonable request and execution of appropriate institutional data use agreements.

## Abbreviations

CXR: Chest Xray
ARF: Acute respiratory failure
ASD: Airspace disease
RALE: Radiographic assessment of lung edema
ARDS: Acute respiratory distress syndrome
ICU: Intensive care unit
CHF: Congestive heart failure
AoCHRF: Acute on chronic hypercapnic respiratory failure
AE-ILD: Acute exacerbation of interstitial lung disease
