# Supplementary material for: Quantifying diffuse airspace disease on portable chest radiographs in acute respiratory failure using the RALE score
Source: Intensive Care Med Exp. 2026 Jul 28;14:102. doi: 10.1186/s40635-026-00953-x (PMC13415720; doi:10.1186/s40635-026-00953-x)
Supplement: Supplementary file 1 — Additional file 1. [file 40635_2026_953_MOESM1_ESM.docx]

**Quantifying Diffuse Airspace Disease on Portable Chest Radiographs in Acute Respiratory Failure Using the RALE Score**

Authors: Vanessa Gipson^1^, Hafiz Qurashi^2^, Pranav Jain^3^, Neha Singh^3^, Kyle Inman^3^, Alicia N. Rizzo^3^, Jacob Sinopoli^1^, Taaha Mirza^4^, Rimsha Ali^4^, Nima Naghshtabrizi^3^, Hanna Bobrysheva^4^, Nanditha Venkatesan^1^, Konstantin Golubykh^4^, Niall Prendergast^3^, Angela Hendricks^5^, John-Paul Oliveria^5^, Joshua Galanter^5^, Julia Cluceru^5^, Mohammadreza Negahdar^5^, Caitlin Schaefer^3^, Melissa Saul^3^, Robin Joyce^6^, Kevin Mitchel^6^, Ellen Hughes^6^, Bryan J. McVerry^7,8,9^, Seyed Mehdi Nouraie^3,9^, Georgios D. Kitsios^3,9^

**Institutions/affiliations:**

1 Internal Medicine Residency Program, University of Pittsburgh, Pittsburgh, Pennsylvania, USA.

2 Division of General Internal Medicine, University of Pittsburgh, Pittsburgh, Pennsylvania, USA.

3 Division of Pulmonary, Allergy and Critical Care Medicine, University of Pittsburgh, Pittsburgh, Pennsylvania, USA.

4 Internal Medicine Residency Program, UPMC Central Pa, Harrisburg, Pennsylvania, USA.

5 Genentech, Inc., South San Francisco, California, USA.

6 Computer Vision Group, Veytel Inc., Pittsburgh, Pennsylvania, USA.

7 VA Pittsburgh Healthcare System, Pittsburgh, Pennsylvania, USA.

8 Department of Critical Care Medicine, University of Pittsburgh School of Medicine, Pittsburgh, Pennsylvania, USA.

9 Acute Lung Injury and Infection Center of Excellence, University of Pittsburgh, Pittsburgh, Pennsylvania, USA.

Corresponding author:

Georgios D. Kitsios, MD, PhD

Assistant Professor of Medicine

Division of Pulmonary, Allergy, Critical Care, and Sleep Medicine

University of Pittsburgh Medical Center

Address: UPMC Montefiore Hospital, NW628, 3459 Fifth Avenue, Pittsburgh, PA 15213

Email: [kitsiosg@upmc.edu](mailto:kitsiosg@upmc.edu)

[**Table of Contents**](#_Toc223093501)

Supplementary Figure 1: **Inter-Rater Agreement for ARF Clinical Subtype Classification**

[Supplemental Figure 2. Representative examples of radiographic penetration levels and poor image quality.](#_Toc223093505)

[Supplemental Figure 3. RALE on the index CXR by ASD status (Absent vs Present).](#_Toc223093502)

[Supplemental Figure 4. Calibration of RALE-based prediction of ASD presence using the index CXR (10-fold cross-validation).](#_Toc223093503)

[Supplemental Figure 5. Predictive values across ASD prevalence for RALE operating points on the index CXR.](#_Toc223093504)

[Supplemental Table 1. Adjudication criteria for ARDS risk factors.](#_Toc223093506)

[Supplemental Table 2. Confusion matrices for cohort-derived RALE operating points on the index CXR for discriminating ASD presence.](#_Toc223093507)

[Supplemental Table 3. Operating characteristics of cohort-derived index RALE thresholds for discriminating diffuse versus limited airspace disease among ASD-positive patients (N=700).](#_Toc223093508)

[Supplemental Table 4. Image-level associations of image acquisition features with RALE magnitude across all CXRs (N=4,258 images).](#_Toc223093509)

[Supplemental Table  5. Effect of age and body mass index on RALE-based discrimination and RALE magnitude (index CXR).](#_Toc223093510)

**Supplemental Figure 1. Inter-Rater Agreement for ARF Clinical Subtype Classification**

Proportion of cases with unanimous agreement among consensus committee members for each ARF clinical subtype. Values represent the percentage of patients for whom all committee members independently assigned the same subtype classification prior to consensus discussion. These agreement statistics were derived from 331 participants for whom survey-based consensus adjudication meetings were conducted, as described in the published adjudication methodology (Kitsios et al., Chest Critical Care 2026). Airway controls (92.9%), acute exacerbation of interstitial lung disease (AE-ILD, 87.5%), and ARDS (84.5%) demonstrated the highest inter-rater agreement. Other/Multifactorial classification showed the lowest agreement (16.7%), consistent with the inherently ambiguous nature of this category. Red dashed line indicates 80% agreement threshold.

Supplemental Figure 2. Representative examples of radiographic penetration levels and poor image quality.
Top row: Examples of frontal portable chest radiographs demonstrating graded radiographic penetration (adequacy) assessed by identifying the deepest visible spinal landmark, All vertebral bodies(All), diaphragm, heart, carina, or thoracic inlet.

Bottom row: Representative examples of poor image quality illustrating technical limitations, including suboptimal inspiratory effort, patient rotation, and superimposed external artifacts. Images are provided for illustrative purposes to demonstrate operational definitions used in the study.


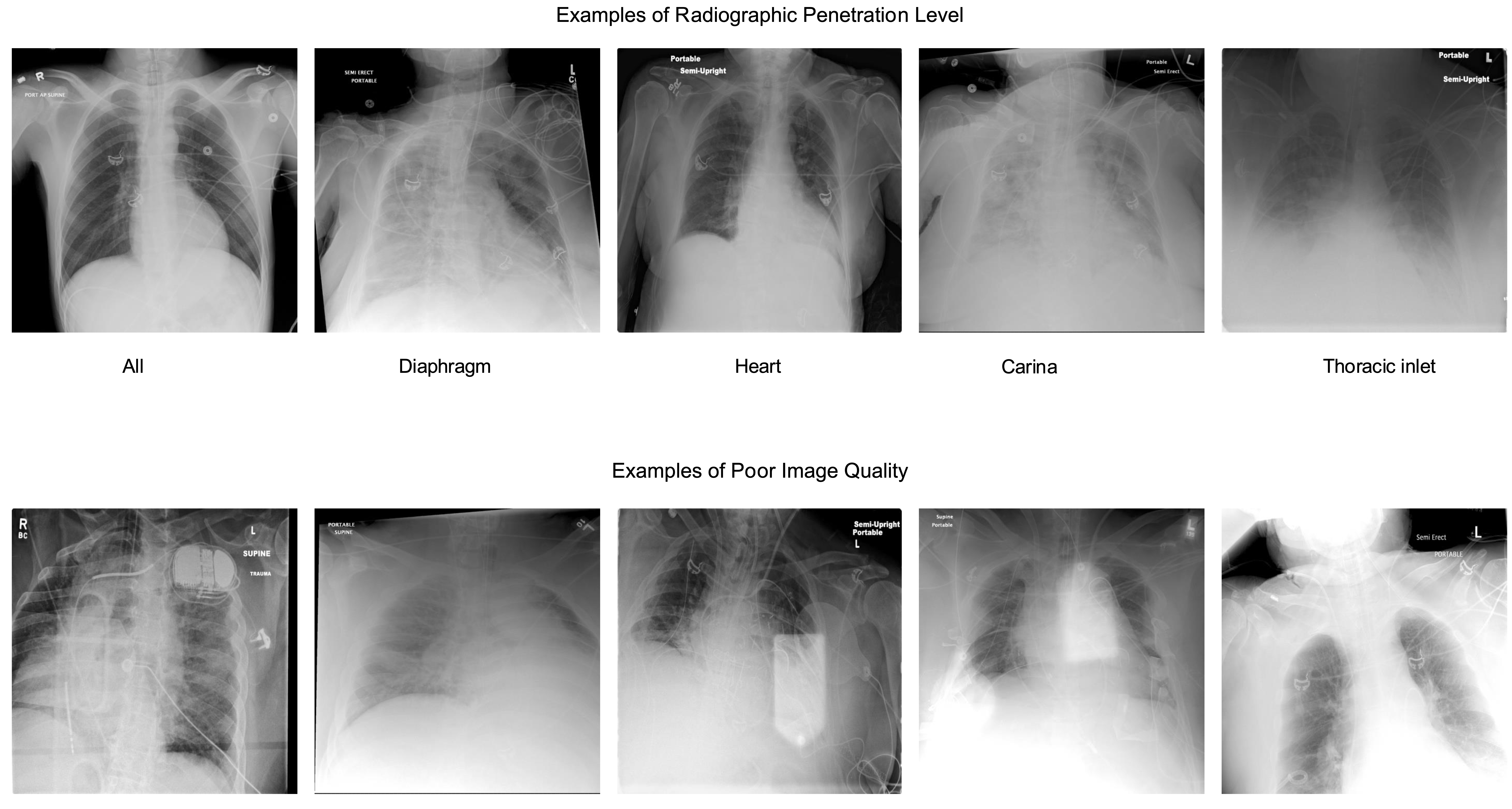


# Supplemental Figure 3. RALE on the index CXR by ASD status (Absent vs Present).

Violin/boxplots show the distribution of RALE scores (0–48) for ASD Absent (n=114) and ASD Present (n=700); points represent individual patients. Groups were compared using the Wilcoxon rank-sum test (p<0.0001).


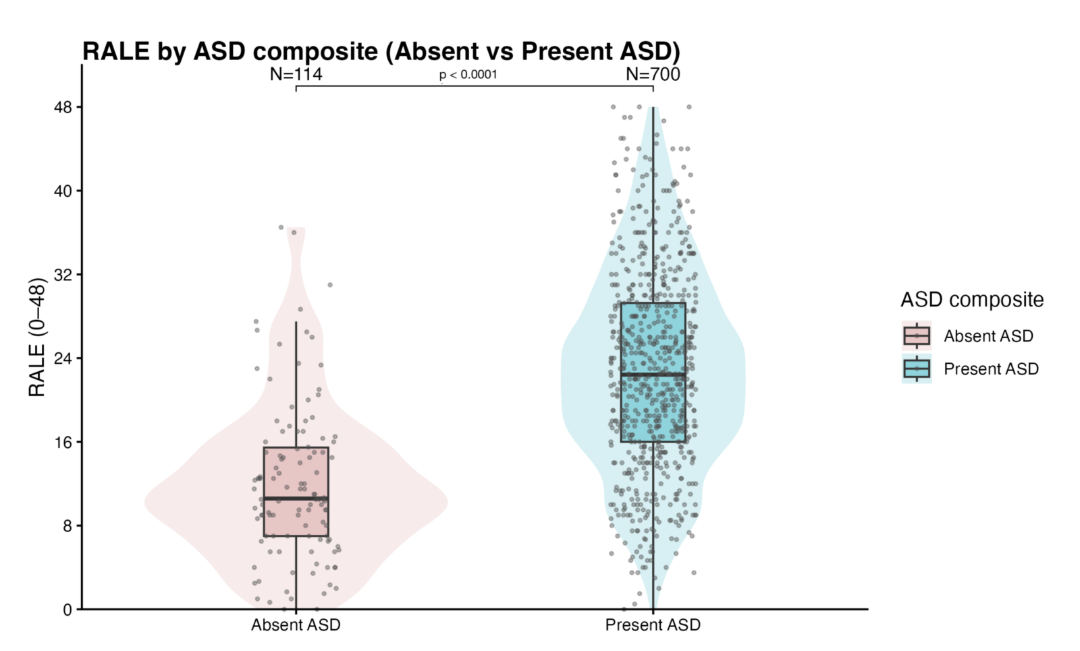


# Supplemental Figure 4. Calibration of RALE-based prediction of ASD presence using the index CXR (10-fold cross-validation).

The x-axis shows out-of-fold predicted probabilities of ASD presence from the RALE-based model on the index CXR, and the y-axis shows the observed proportion of ASD presence within groups of predictions. Point size reflects the number of subjects per group; the solid line depicts the smoothed calibration curve and the dashed 45° line indicates perfect calibration. Calibration-in-the-large (intercept), calibration slope, and Brier score are shown.


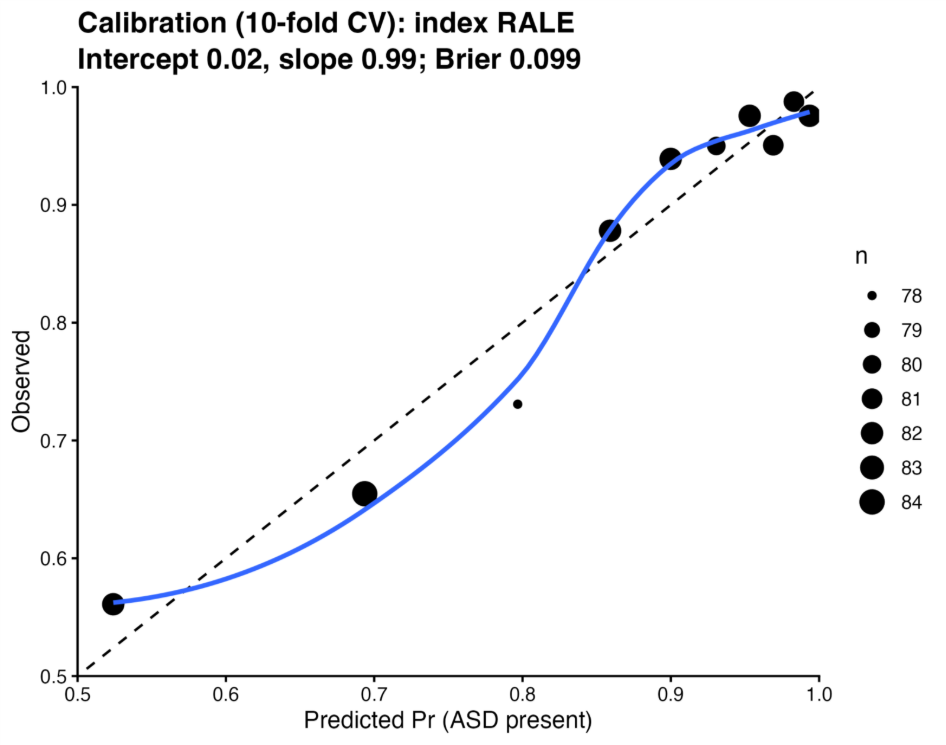


# Supplemental Figure 5. Predictive values across ASD prevalence for RALE operating points on the index CXR.

Curves show expected positive predictive value (PPV; left) and negative predictive value (NPV; right) across a range of ASD prevalence, holding sensitivity and specificity fixed at the values observed for the two cohort-derived RALE cutoffs on the index CXR (rule-out cutoff targeting sensitivity ≥95% and the Youden cutoff). The vertical dashed line denotes the ASD prevalence in this cohort (86%; 700/814), and points indicate the corresponding PPV and NPV at that prevalence.


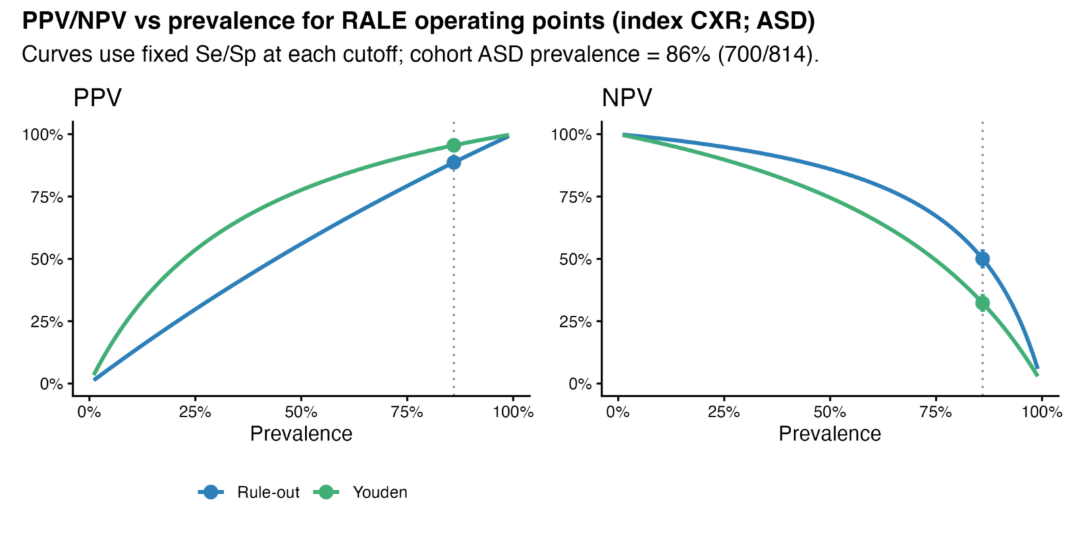


Supplemental Table 1. Adjudication criteria for ARDS risk factors. 
Risk factors were classified as direct (primary pulmonary insults) or indirect (extrapulmonary conditions associated with systemic inflammation and secondary lung injury) based on prespecified registry definitions. The table lists each risk factor and the operational adjudication criteria used to assign a category. Abbreviations: WBC, white blood cell; TRALI, transfusion-related acute lung injury.

| **Risk Factor** | **Category Of Injury** | **Adjudication Criteria** |
| --- | --- | --- |
| **Pneumonia** | Direct | Clinical Diagnosis supported by chest imaging demonstrating airspace opacities plus >1 of the following: purulent sputum, abnormal temperature (<36°C or >38°C), or abnormal WBC (>12,000/µL, <4,000/µL, or >10% bands) |
| **Aspiration (Macro-Aspiration)** | Direct | Witnessed or documented aspiration event, or retrieval of gastric contents from the oropharynx, endotracheal tube, or bronchial tree based on presenting history or procedural documentation (intubation or bronchoscopy). |
| **Inhalational Injury** | Direct | Documented exposure to inhaled substances known to cause lung injury, including vaping, illicit drug inhalation, or smoke inhalation from fire exposure. |
| **Pulmonary Contusion** | Direct | Imaging evidence of focal pulmonary opacities in the setting of blunt thoracic trauma or following prolonged cardiopulmonary resuscitation. |
| **Pulmonary Vasculitis** | Direct | Diagnosis supported by positive autoimmune serologies and/or lung biopsy findings consistent with vasculitis |
| **Drowning** | Direct | Aspiration of salt or freshwater associated with a documented drowning event. |
| **Non-Pulmonary Sepsis** | Indirect | Sepsis defined as infection with associated end-organ dysfunction originating from a non-pulmonary source. |
| **Major Trauma** | Indirect | Blunt or penetrating trauma involving the chest or abdomen, or presence of large bone fractures. |
| **Pancreatitis** | Indirect | Clinical history consistent with acute pancreatitis, elevated serum lipase, and characteristic CT abdomen findings. |
| **Severe Burns** | Indirect | Partial- or full-thickness burns involving ≥20% total body surface area. |
| **Non-Cardiogenic Shock** | Indirect | Hypotension and tissue hypoperfusion due to non-cardiogenic causes (e.g., vasoplegic shock from acute liver failure) |
| **Drug Overdose** | Indirect | Overdose involving opiates, cocaine, or amphetamines |
| **Massive Transfusion** | Indirect | Transfusion of >4 units of packed red blood cells within 24 hours |
| **TRALI** | Indirect | Transfusion of fresh frozen plasma followed by acute lung injury in the absence of another identifiable lung injury risk factor |

# Supplemental Table 2. Confusion matrices for cohort-derived RALE operating points on the index CXR for discriminating ASD presence.

Counts show true positives (TP), false positives (FP), false negatives (FN), and true negatives (TN) for two integer RALE cutoffs (RALE ≥7 and RALE ≥17) applied to the index CXR. ASD status is the reference standard (ASD present = reference positive; ASD absent = reference negative).

| **Threshold** | **RALE test result** | **ASD present (reference +)** | **ASD absent (reference -)** |
| --- | --- | --- | --- |
| Rule-Out (RALE ≥ 7) | Test + | TP = 672 | FP = 86 |
| Rule-Out (RALE ≥ 7) | Test - | FN = 28 | TN = 28 |
| Youden (RALE ≥ 17) | Test + | TP = 511 | FP = 24 |
| Youden (RALE ≥ 17) | Test - | FN = 189 | TN = 90 |

# Supplemental Table 3. Operating characteristics of cohort-derived index RALE thresholds for discriminating diffuse versus limited airspace disease among ASD-positive patients (N=700).

Sensitivity, specificity, positive predictive value (PPV), negative predictive value (NPV), positive likelihood ratio (LR+), and negative likelihood ratio (LR−) are shown for two integer RALE thresholds applied as RALE ≥ cutoff on the index CXR. The Youden cutoff maximizes Youden’s J; the rule-out cutoff is the lowest integer RALE achieving sensitivity ≥0.95. Values are point estimates with 95% confidence intervals.

| **Threshold** | **RALE cutoff** | **Sensitivity (95% CI)** | **Specificity (95% CI)** | **PPV (95% CI)** | **NPV (95% CI)** | **LR+** | **LR−** |
| --- | --- | --- | --- | --- | --- | --- | --- |
| Youden | 20 | 0.78 (0.74–0.82) | 0.66 (0.60–0.71) | 0.78 (0.74–0.82) | 0.67 (0.61–0.73) | 2.33 | 0.33 |
| Rule-out (Sens ≥95%) | 12 | 0.95 (0.92–0.97) | 0.29 (0.23–0.34) | 0.67 (0.63–0.71) | 0.80 (0.70–0.87) | 1.34 | 0.17 |

**Supplemental Table 4. Image-level associations of image acquisition features with RALE magnitude across all CXRs (N=4,258 images).**

Multivariable linear mixed-effects model with random intercepts by SubjectID. β coefficients are in RALE points (natural scale) with 95% confidence intervals. Penetration is suboptimal vs adequate; overall image quality is poor vs good. Artifact burden is modeled as the per-image count of artifact annotations (β shown per one additional annotation).

| **Predictor** | **β (RALE points)** | **95% CI** | **p-value** |
| --- | --- | --- | --- |
| **Penetration** |  |  |  |
| Penetration: Suboptimal (vs Adequate) | 3.29 | 2.50 – 4.08 | <0.0001 |
| **Artifacts** |  |  |  |
| Artifacts (per additional annotation) | -0.10 | -0.27 − 0.07 | 0.26 |
| **Quality** |  |  |  |
| Quality: Poor (vs Good) | -0.07 | -1.39 − -1.24 | 0.91 |

Supplemental Table 5. Effect of age and body mass index on RALE-based discrimination and RALE magnitude (index CXR). 
Panel A: Logistic regression for ASD present vs ASD absent on the index CXR comparing a RALE-only model to RALE plus age and BMI; the ΔAUC p-value is from a paired DeLong test. AUC indicates area under the receiver operating characteristic curve; 95% CIs are DeLong intervals. 
Panel B: Linear regression with RALE (points) as the dependent variable; β denotes the mean change in RALE per 1-year increase in age and per 1 kg/m² increase in BMI, with cluster-robust standard errors by SubjectID.

**Panel A. Discrimination (AUC)**

| **Model** | **AUC (95% CI)** | **ΔAUC p-value** |
| --- | --- | --- |
| RALE only | 0.81 (0.77–0.85) |  |
| RALE + age + BMI | 0.82 (0.78–0.86) | 0.154 |

**Panel B. Association with RALE magnitude**

| **Predictor** | **β (RALE points)** | **95% CI** | **p-value** |
| --- | --- | --- | --- |
| Age (per year) | -0.02 | -0.07–0.02 | 0.263 |
| BMI (per kg/m²) | 0.25 | 0.19–0.32 | <0.001 |
